# Single Fetal Demise at 10 - 14 Weeks of Monochorionic and Dichorionic Twin Pregnancy

**DOI:** 10.14740/jocmr2479w

**Published:** 2016-02-27

**Authors:** Shunji Suzuki

**Affiliations:** Department of Obstetrics and Gynecology, Japanese Red Cross Katsushika Maternity Hospital, 5-11-12 Tateishi, Katsushika-ku, Tokyo 124-0012, Japan. Email:czg83542@mopera.ne.jp

**Keywords:** Fetal demise, First trimester, Monochorionic twin pregnancy

## Abstract

**Background:**

We examined the perinatal outcomes in cases of at least one fetal demise in monochorionic and diamniotic twin pregnancies.

**Methods:**

We reviewed the obstetric records of all Japanese twin pregnancies managed beyond 9 weeks’ gestation at Japanese Red Cross Katsushika Maternity Hospital between 2008 and 2014.

**Results:**

The incidence in the monochorionic twin pregnancies was significantly higher than that in the dichorionic twin pregnancies (8.3% vs. 2.6%, odds ratio: 3.40, 95% confidence interval: 1.5 - 7.6, P < 0.01). Of these, 60.0% were diagnosed as fetal demise of both fetuses at the same time (vs. dichorionic twin pregnancy: odds ratio: 12.0, 95% confidence interval: 1.3 - 120, P = 0.04). The rate of “vanishing twin” in cases of at least one fetal demise at 10 - 14 weeks’ gestation in the monochorionic twin pregnancies was significantly lower than that in the diamniotic twin pregnancies (30.0% vs. 88.9%, odds ratio: 0.05, 95% confidence interval: 0.01 - 0.53, P = 0.01).

**Conclusion:**

The incidence of fetal demise and the influence on the co-twin in monochorionic twin pregnancy is greater than those in dichorionic twin pregnancy.

## Introduction

In an earlier examination by Landry and Weingold [1], ultrasound during the first trimester demonstrated that the incidence of twins seems to be greater than previously presumed. For example, twins have been observed to occur in 12% of all spontaneous conceptions; however, only 14% of twin pregnancies survive to term [2]. A portion of first trimester detected twin pregnancies result in spontaneous loss of the entire pregnancy, whereas others are complicated by an early demise of one fetus and deliver at term as singletons as “vanishing twin” [1, 3]. First trimester loss of one fetus in dichorionic twin pregnancy has not been seemed to result in adverse outcomes for the surviving co-twin [3, 4]. In monochorionic twin pregnancies, however, it has been reported to be at increased risk for adverse outcomes associated with twin-twin transfusion syndrome (TTTS) [3, 4].

In this study, we examined the perinatal outcomes in cases of at least one fetal demise in monochorionic and diamniotic twin pregnancies.

## Methods

We reviewed the obstetric records of all Japanese twin pregnancies managed beyond 9 weeks’ gestation at Japanese Red Cross Katsushika Maternity Hospital between 2008 and 2014. The protocol for this analysis was approved by the Ethics Committee of the Japanese Red Cross Katsushika Maternity Hospital. In addition, informed consent for analysis from a retrospective database was obtained from each subject during their hospital visit. Demographic information and the characteristics of twin pregnancy were extracted from patient charts.

The gestational ages of the pregnancies were established by ultrasonographic examination of the fetal crown-rump length at 9 - 10 weeks’ gestation in cases of spontaneous conception and embryo transfer dates when pregnancy was achieved by *in vitro* fertilization. In this study, the gestational ages of fetal demise were diagnosed by ultrasonographic examination of the fetal crown-rump length at 10 - 11 weeks’ gestation or biparietal diameter at 12 - 14 weeks’ gestation. In our institute, chorionicity was determined until the end of 10 weeks of twin pregnancy. In addition, all twin pregnancies were examined using ultrasonography at least once every 2 weeks. When a dead twin was growing, twin reversed arterial perfusion (TRAP) sequence was diagnosed [5].

X^2^ or Fisher’s exact test was used for categorical variables. Odds ratios and 95% confidence intervals were also calculated. Differences with P < 0.05 were considered significant.

## Results

There were 346 dichorionic diamniotic (DD) and 240 monochorionic diamniotic (MD) twin pregnancies managed beyond 9 weeks’ gestation during the study period.

Of the 346 DD twin pregnancies, nine cases (2.6%) resulted in at least one fetal demise at 10 - 14 weeks’ gestation. Of them, one case was diagnosed as fetal demise of both fetuses at the same time. The rest eight cases (88.9%) delivered at term as singletons.

Of the 240 MD twin pregnancies, 20 cases (8.3%) resulted in at least one fetal demise at 10 - 14 weeks’ gestation. The incidence in the MD twin pregnancies was significantly higher than that in the DD twin pregnancies (odds ratio: 3.40, 95% confidence interval: 1.5 - 7.6, P < 0.01). Of these, 12 cases (60.0%) were diagnosed as fetal demise of both fetuses at the same time (vs. DD twin pregnancy: odds ratio: 12.0, 95% confidence interval: 1.3 - 120, P = 0.04). TRAP sequence was diagnosed in two cases (10.0%) of fetal demise at 10 and 16 weeks’ gestation. These two cases of TRAP sequence resulted in fetal demise of the co-twin at 14 and 16 weeks’ gestation. The rest six cases (30.0%) delivered at term as singletons. Therefore, the rate of “vanishing twin” in cases of at least one fetal demise at 10 - 14 weeks’ gestation in the MD twin pregnancies was significantly lower than that in the DD twin pregnancies (30.0 vs. 88.9%, odds ratio: 0.05, 95% confidence interval: 0.01 - 0.53, P = 0.01).

There were no cases complicated by neurological sequelae in these cases of “vanishing twin” based on the follow-up of at least 1 year by the pediatricians of our hospital.

## Discussion

During the second and third trimester of monochorionic pregnancy, single demise has been associated with multiorgan damages in the surviving co-twin such as cerebral ischemic changes [4-7]. However, it has been unclear at which gestational age one fetal demise in monochorionic pregnancy could result in adverse sequelae for the surviving co-twin. In some recent reports [8, 9], early demise in a monochorionic pregnancy has been reported to occur secondary to acute and early TTTS could be an explanation for a portion of cases of unexplained cerebral palsy. Otherwise, early fetal demise can cause serious hemodynamic changes in the surviving co-twin resulting in both fetal demise and cerebral ischemic changes. In this study, the incidence of fetal demise of both fetuses diagnosed at the same time in MD twin pregnancy was significantly higher than that in DD twin pregnancy. The result may indicate the presence of serious feto-placental hemodynamic changes secondary to fetal demise in MD twin pregnancy. Fortunately, there were no cases with neurological sequelae in our “vanishing monochorionic twins”; however, Weiss et al reported a case of fetal demise of one twin at 12 weeks in monochorionic twin pregnancy which resulted in multicystic encephalomalacia in the surviving twin diagnosed using magnetic resonance imaging [8]. Therefore, further large studies in cases of “vanishing monochorionic twin” concerning the possibility of neurological sequelae may be needed.

TRAP sequence has been reported to occur when one twin, lacking a functioning cardiac system (“acardiac twin”), receives blood from the normally developing “pump twin” in monochorionic twin pregnancy [10-13]. The sequence puts the pump twin in the risk of cardiac failure. TRAP sequence can be treated *in utero* using selective bipolar cord coagulation, selective cord occlusion and radiofrequency ablation procedures. In the untreated cases, the “pump twin” has been observed to die in 50-75% of cases. In this study, unfortunately, the therapies *in utero* could not be performed because of the fast time courses. Therefore, a frequent follow-up is necessary using ultrasonograpy in cases of fetal demise in monochorionic twin pregnancy.

### Conclusions

The incidence of fetal demise and the influence on the co-twin in monochorionic twin pregnancy is greater than those in dichorionic twin pregnancy. A frequent follow-up is necessary using ultrasonograpy in cases of fetal demise in monochorionic twin pregnancy.
